# GnRH Agonist Triggering of Ovulation Replacing hCG: A 30-Year-Old Revolution in IVF Practice Led by Rambam Health Care Campus

**DOI:** 10.5041/RMMJ.10300

**Published:** 2017-04-28

**Authors:** Shahar Kol, Ofer Fainaru

**Affiliations:** IVF Unit, Rambam Health Care Campus, Haifa, Israel; and The Ruth and Bruce Rappaport Faculty of Medicine, Technion–Israel Institution of Technology, Haifa, Israel

**Keywords:** GnRH agonist, GnRH antagonist, hCG, *in vitro* fertilization, ovarian hyperstimulation syndrome, ovulation triggering

## Abstract

Final oocyte maturation is a crucial step in *in vitro* fertilization, traditionally achieved with a single bolus of human chorionic gonadotropin (hCG) given 36 hours before oocyte retrieval. This bolus exposes the patient to the risks of ovarian hyperstimulation syndrome (OHSS), particularly in the face of ovarian hyper-response to gonadotropins. Although multiple measures were developed to prevent OHSS, gonadotropin-releasing hormone (GnRH) agonist triggering is now globally recognized as the best approach to achieve this goal. The first report on the use of GnRH agonist as ovulation trigger in the context of OHSS prevention came from Rambam Health Care Campus, Haifa, Israel and appeared in 1988. This review details the events that culminated in worldwide acceptance of this measure and describes its benefit in the field of assisted reproductive technology.

## INTRODUCTION

The first successful human *in vitro* fertilization (IVF) treatment was reported in 1978.^1^ It was achieved by harvesting an oocyte by laparoscopy in a natural cycle. This approach was associated with very low chances of live birth and was soon replaced by strategies that are more efficient. A major advantage was to stimulate the ovaries with gonadotropins in order to harvest more oocytes. In addition, it became necessary to control the time of ovulation, so oocyte retrieval could be scheduled to acceptable working hours. In a natural ovulatory cycle, a sharp rise in luteinizing hormone (LH) (and follicle-stimulating hormone, FSH) serum level is the biochemical trigger of a cascade of events resulting in ovulation. Since LH was not available, a surrogate molecule, human chorionic gonadotropin (hCG), was used for this purpose. In addition, since spontaneous ovulation led to treatment cancellations, its hormonal inhibition was sought in order to prevent premature luteinization. Since gonadotropin-releasing hormone (GnRH) agonists were developed at that stage, their ability to downregulate pituitary LH and FSH secretion helped increase treatment efficiency.^2^ However, at the same time, the combination of ovarian stimulation by gonadotropins after GnRH agonist-induced pituitary downregulation and hCG as final oocyte maturation trigger led to a sharp increase in the risk of ovarian hyperstimulation syndrome (OHSS). Although multiple approaches were suggested to prevent OHSS, they all fell short of achieving this goal.

## GnRH AGONIST TRIGGER IN THE CONTEXT OF OHSS PREVENTION

The first report on the use of a GnRH agonist as ovulation trigger in the context of OHSS prevention came from the Rambam Health Care Campus (Rambam), Haifa, Israel and appeared in 1988 as an abstract.^3^ Soon after, a full paper was published, underscoring the concept that a bolus of GnRH agonist is able to trigger an adequate mid-cycle LH/FSH surge, resulting in oocyte maturation and pregnancy. In addition, experience with eight patients with exaggerated response suggested that it might prevent the clinical manifestation of OHSS.^4^ A full review of the hormonal events after GnRH agonist trigger followed.^5^ As more experience was gained with GnRH agonist triggering, it became apparent that conducting a randomized controlled study, comparing hCG and GnRH agonist triggers in high-risk patients, would be unethical. Therefore, a case–control study was published in an effort to describe the unparalleled strength of this modality in comparison to other strategies used to prevent OHSS.^6^ The use of intravenous albumin around the time of oocyte retrieval was also suggested as a major intervention to prevent OHSS; however, our experience, as well as that of others, led to abandonment of this approach.^7^

## PRE-GnRH ANTAGONIST ERA

Importantly, the IVF community was reluctant to adopt the GnRH agonist trigger in the context of OHSS prevention, since up to the year 2000 GnRH agonists were used solely to prevent premature LH rise and luteinization by pituitary downregulation in most IVF cycles. Naturally, under these protocols, a GnRH agonist could not be used as a trigger. In addition, the experience at Rambam of total elimination of OHSS was met with skepticism and disbelief. In order to allow a GnRH agonist trigger, a GnRH analog-free protocol must be used, with increased risk of cycle cancellation due to premature LH rise and luteinization. On the other hand, a GnRH agonist trigger was used in “hyper-responder” patients, in whom high serum concentrations of gonadotrophin surge-attenuating factor (GnSAF) are found. The GnSAF decreases the risk of premature luteinization by its action on endogenous GnRH secretion pattern.^8^,^9^ Therefore, in IVF patients at high risk of OHSS, the risk of premature luteinization and cycle cancellation is relatively low, even if GnRH agonists are not used for pituitary downregulation.

## AT THE TURN OF THE CENTURY

During the last decade of the twentieth century, intensive pharmaceutical efforts reached the target of producing GnRH antagonists with proven clinical activity and few side effects.^10^ These products were used in conjunction with recombinant FSH preparations. The first pregnancy in the world with such a combination was achieved at Rambam.^11^ More importantly, GnRH antagonists allowed for the use of GnRH agonists as ovulation trigger. Since the last antagonist dose is given many hours before the GnRH agonist trigger dose, it seems plausible that the agonist trigger under these circumstances can elicit an adequate LH surge to secure final oocyte maturation. The first proof-of-concept study along these lines was performed at Rambam.^12^ The report described the use of 0.2 mg triptorelin (Decapeptyl, Ipsen Pharma Biotech, Signes, France) to trigger ovulation in eight patients who underwent controlled ovarian hyperstimulation with recombinant FSH and concomitant treatment with the GnRH antagonist ganirelix (Orgalutran, N.V. Organon, BH OSS, The Netherlands) for the prevention of premature LH surges. All patients were considered to have an increased risk for developing OHSS. After GnRH agonist injection, endogenous serum LH and FSH surges were observed with median peak values of 219 and 19 IU/L respectively, measured 4 h after injection. These values are comparable to those described for women that were not exposed to GnRH antagonist treatment before the GnRH agonist trigger. The mean number of oocytes obtained was 23.4±15.4, of which 83% were mature (metaphase II). None of the patients developed any signs or symptoms of OHSS. The ability of the GnRH agonist to trigger final oocyte maturation after co-treatment with the GnRH antagonist during ovarian hyperstimulation for *in vitro* fertilization was subsequently confirmed.^13^

## MECHANISM OF OHSS PREVENTION

Although the efficient capacity of GnRH agonist triggering to prevent OHSS was evident, the mechanism by which it works was not clear. To shed more light on this aspect, luteal activity post GnRH agonist trigger was examined. Specifically, corpus luteum function was assessed by measuring luteal phase levels of inhibin A and pro-αC, peptides that reflect luteal phase hormonal activity. These peptides were measured in a small prospective randomized trial, after controlled ovarian hyperstimulation with FSH and GnRH antagonist. Following triggering of final oocyte maturation with either hCG (*n*=8) or GnRH agonist (*n*=8), blood was collected every 2–3 days during the luteal phase.^14^ Levels of inhibin A, pro-αC, estradiol, and progesterone were significantly lower from day 4 to day 14 after triggering final oocyte maturation by GnRH agonist compared with hCG. These results confirm that triggering final oocyte maturation with GnRH agonist instead of hCG in IVF cycles dramatically decreases luteal levels of inhibins, reflecting significant inhibition of corpus luteum function. Following this publication, the term “OHSS-free clinical environment” was coined,^15^ later repeated by others.^16^

## WORLDWIDE DISSEMINATION

The first (and probably last) randomized controlled study comparing outcome after GnRH agonist and hCG triggers in OHSS high-risk patients was published in 2008.^17^ None of the patients in the study group developed any form of OHSS, compared with 31% (10/32) of the patients in the control group. Such a big difference left any further similar studies unethical to conduct, and convinced the international fertility community that the GnRH agonist trigger is the most efficient means to prevent OHSS. Soon, a Cochrane review documented GnRH agonist triggering as a well-established mode of OHSS prevention.^18^ Similarly, professional organizations echoed the same message: “… strong evidence that the use of a GnRH agonist trigger results in a significant reduction in the development of OHSS.”^19^ Finally, as of 2013, more than one-third of IVF cycles in Europe were triggered with GnRH agonist.^20^

The growing interest in GnRH agonist triggering also prompted the creation of a special interest group, “The Copenhagen GnRH Agonist Triggering Workshop Group,” that was instrumental in expediting the “change of practice” from hCG to GnRH agonist trigger.^21^

## SIDE BENEFITS

During the follicular phase, the dominant follicle acquires the ingredients and maturation stage for ovulation. The pituitary, firing a biochemical trigger (LH and FSH surges), controls the cue for ovulation. Luteinizing hormone homology and a relatively easy manufacturing process makes hCG an excellent molecule to be used in triggering ovulation. Unlike the LH/FSH surge, which terminates 48 hours after its onset, hCG activity spans long into the luteal phase. This supra-physiologic LH-like activity overstimulates the corpora lutea, leading to high serum estradiol levels, which in turn decreases endogenous LH secretion. Luteal phase defect follows, which makes luteal phase support mandatory in order to maintain a receptive endometrium. An effort to introduce recombinant LH as a trigger was published in 2001 by the European Recombinant LH Study Group,^22^ but the project was terminated due to high cost and low success rate.

Although the role of the FSH mid-cycle surge is not fully explored, it is known to promote LH receptor formation in luteinizing granulosa cells, nuclear maturation (i.e. resumption of meiosis), and cumulus expansion.^23^,^24^ Previously, we have been relying solely on LH activity-dependent (hCG) triggering of final oocyte maturation, assuming that the natural mid-cycle FSH surge is biologically redundant. However, several authors have reported an increase in oocyte yield following the GnRH agonist trigger compared with the routine hCG trigger.^25^ It cannot be ruled out that the added FSH surge contributes to this difference. A simple way to prompt FSH surge is to administer GnRH agonist.

## MANIPULATING LUTEAL PHASE SUPPORT

If OHSS is not a concern, GnRH agonist can be followed by two boluses of hCG (1,500 IU each) for luteal rescue, without any additional progesterone-based luteal support.^26^ Thus, the triggering (high-dose) property of hCG is dissected from its luteal supportive properties (low-dose), which imitates normal physiology more closely, and yields high progesterone levels during the implantation window. In OHSS high-risk patients high-dose progesterone and estradiol may offer a good clinical outcome with almost total elimination of OHSS.^17^ Another option is to support the luteal phase with a single bolus of low-dose hCG (1,500 IU instead of the usual trigger dose of 6,500 IU).^25^

Previously, follicular phase “coasting” in the “long” GnRH agonist downregulation protocol has been suggested as a strategy for OHSS prevention. Coasting seeks to induce partial atresia of the developing follicles by withholding gonadotropin stimulation, while monitoring estradiol levels, considered to reflect theca—and granulosa—cell function. Using this strategy, hCG trigger is administered when estradiol levels drop below a certain cut-off level, reflecting partial demise of the developing follicles, decreasing the burden of multiple corpora lutea formation that follows. To individualize luteal phase support in OHSS high-risk patients, having a fresh transfer after GnRH agonist trigger, the same principle that holds for follicular phase coasting might be valid during the luteal phase—in other words, monitoring progesterone levels, and administering the hCG luteal phase rescue bolus (1,500 IU) when progesterone levels drop significantly. This approach was implemented and published recently,^27^ taking progesterone level of 30 nmol/L as a threshold level for hCG administration.

As we gain more experience with GnRH agonist trigger, we have documented rare cases in which luteolysis does not occur, and intensive endogenous luteal function is maintained, leaving any additional exogenous support redundant and exposing the patient to OHSS risk.^28^,^29^

## SUMMARY

This short review describes the events that were initiated at Rambam and led to a profound change in IVF practice. Over the last 30 years the research and clinical experience led by Rambam IVF has reached worldwide acceptance, as more and more cycles are now triggered with GnRH agonists. The primary advantage of this elegant triggering mode is improvement in IVF safety, as it has led to almost total elimination of OHSS, a potentially lethal complication of ovarian stimulation.^30^

## Abbreviations

FSH: follicle-stimulating hormone
GnRH: gonadotropin-releasing hormone
GnSAF: gonadotrophin surge-attenuating factor
hCG: human chorionic gonadotropin
IVF: *in vitro* fertilization
LH: luteinizing hormone
OHSS: ovarian hyperstimulation syndrome
Rambam: Rambam Health Care Campus
